# Surveying Adolescents During a Pandemic: Comparison of Adolescents Recruited via Social Media vs. Schools

**DOI:** 10.1007/s11121-023-01621-2

**Published:** 2023-12-01

**Authors:** Jennifer B. Unger, Jane Steinberg, Robert Vos, Daniel W. Soto, Larisa Albers, Christopher J. Rogers

**Affiliations:** 1 University of Southern California, Los Angeles, CA USA; 2 California State University, Northridge, Northridge, CA USA

## Abstract

School-based surveys of adolescents can be logistically difficult and exclude students who do not attend school. Social media recruitment could be a promising strategy to recruit representative samples of adolescents. However, few studies have compared adolescent survey data collected via different methodologies. Our team was conducting a school-based survey when the COVID-19 pandemic closed all schools, necessitating a shift to online adolescent recruitment. To achieve our goal of obtaining a sample of high school students throughout California, we placed ads on social media. We compared the adolescents recruited in schools with those recruited on social media on demographic characteristics, mental health, and substance use. The sample of students recruited in schools (*N* = 737) and adolescents recruited via social media (*N* = 953) did not differ significantly on gender or substance use. However, compared with school-based recruitment, social media recruitment yielded a higher proportion of boys, whites, and Asians and a lower proportion of girls, Hispanic/Latinx adolescents, and those who spoke other languages at home. The social media sample had significantly higher levels of depression and anxiety symptoms and perceived stress than the school-based sample. Results indicate that social media can be useful for recruiting adolescents for survey research, especially if strategies such as Spanish-language social media ads are used to recruit and consent Hispanic/Latinx adolescents and those with non-English-speaking parents. This method could potentially replace school-based surveys in cases where schools are unwilling to participate in research, or it could be used to supplement school-based samples. Advantages and disadvantages of both methods are discussed.

School-based survey data collection is a common method of obtaining information about adolescent risk behaviors. Schools provide a setting where multiple students can be recruited simultaneously, and the school environment is conducive to paper and online surveys. However, school-based research has several limitations. First, schools receive numerous requests to participate in research and cannot accommodate them all (Esbensen et al., 2008; Plummer et al., 2014). Second, school-based research excludes students who are not in the classroom on the day of the survey, including those who are frequently truant, have dropped out of school, are attending alternative programs, or are home-schooled (Weitzman et al., 2003). Third, adolescents who engage in risky behavior and/or have chaotic family lives might be less likely to return parental consent forms, potentially biasing school-based samples (Anderman et al., 1995; Liu et al., 2017; Unger et al., 2004). Therefore, it is important to identify efficient and effective recruitment methods that can yield large, representative samples of adolescents.

Recruitment via social media could be an effective alternative to in-person recruitment in schools. Because most adolescents use social media, and because social media recruitment does not require in-person staff visits and the use of instructional time in school, social media could be a more efficient way to assemble large, representative samples for population-based studies. However, it is unclear whether social media recruitment produces samples that are representative of the underlying population and/or the school population.

Several studies have compared social media recruitment with other strategies to recruit adolescents. One study (Moreno et al., 2017) compared school- and clinic-based recruitment with social media recruitment for a physical activity monitoring study and found that in-person recruitment yielded more participants and was more efficient in terms of staff time and monetary expense per participant recruited. However, this was a high-intensity study that involved extensive physical activity monitoring. It is unclear whether findings would be similar for a brief survey. Another study (Gu et al., 2016) found that a mailed postcard with a QR code outperformed social media posts for recruiting rural adolescents. A study of sexual and gender minority youth (Stern et al., 2020) found that social media recruitment produced a more racially and ethnically diverse sample than the Youth Risk Behavior Surveillance Survey, which uses school-based recruitment. However, no known studies have compared the characteristics of adolescents recruited via social media with those of adolescents recruited in schools.

When the COVID-19 pandemic abruptly closed schools while we were in the process of recruiting a sample of adolescents, we switched from school-based recruitment to social media recruitment. We compared the demographic characteristics, mental health, and substance use of adolescents recruited via social media with those of adolescents recruited in schools. Because students who engage in risky behaviors and/or have mental health problems might be more likely to be absent from school and more likely to use social media (Vannucci et al., 2020; Vidal et al., 2020), we hypothesized that the adolescents recruited through social media would have a higher prevalence of mental health problems and substance use compared to those recruited in schools. We did not have a priori hypotheses about demographic differences in the two samples. We also compared the samples from both recruitment methods to the overall demographics of adolescents in the same state to determine which recruitment method produced the most representative sample.

## Method

### School-Based and Social Media Recruitment

The CalTeens project (Rogers et al., 2022) was designed to assess the association between proximity to cannabis retail outlets and cannabis use among California adolescents. We planned to recruit a representative statewide sample of California adolescents in high schools throughout the state. Details about the recruitment procedures are described by Rogers et al. (2022). In brief, we began by categorizing all California public high schools into nine strata based on their proximity to cannabis retailers (tertiles) and neighborhood socioeconomic status (tertiles). We were in the process of recruiting high schools in each of these nine strata in the fall of 2019 and the spring of 2020, when the COVID-19 pandemic closed all public schools in California in March of 2020. Therefore, we pivoted to a social media recruitment strategy. Targeted social media ads were placed on Facebook and Instagram using TrialPromoter (Reuter et al., 2016), a web-based tool that repeatedly posts ads on social media to recruit participants into clinical trials. The ads invited California teens to participate in a survey of attitudes and behaviors for a $20 gift card incentive. When social media users clicked on the ad, they were directed to a website to provide their contact information to obtain informed consent. If a parent of a teen clicked on an ad, they were directed to a website to provide parental consent and provide their child’s contact information so we could invite their child. We conducted crosschecks of names, contact information (including phone and address), and IP addresses to verify that participants lived in California and to prevent individuals from completing the survey more than once. Addresses were validated by searching on Google Maps to confirm that the address was a residence and by searching online reverse address databases such as Spokeo and PeopleFinders. We also contacted prospective participants and/or their parents by telephone to confirm their identity and address when necessary. Responses were excluded if respondents were not 14–17 years of age, did not provide a valid residential address, had the same IP address, email, or phone number as a previous participant, or provided a voice over internet protocol (VOIP) phone number rather than a valid cellular or landline phone number.

### Informed Consent

Because the survey asked about sensitive topics and was not anonymous, our IRB required that we obtain parental consent and youth assent. For the school-based recruitment, we sent paper consent forms home with the students for their parents to sign. Students who obtained parental consent were invited to provide youth assent and participate in the survey. For the social media recruitment, when an adolescent clicked on the social media link, they arrived at a study website that asked for the adolescent’s and parent’s contact information. We emailed the parent with a link to the consent form. If the parent signed the consent form electronically, we emailed the student with a link to the assent form and survey.

### Final Analytic Sample

In the school-based recruitment, 1297 students were approached in classrooms, 904 (70%) provided parental consent. Of those with parental consent, 819 (91%) also provided student assent. Of those, 737 (90%) completed the survey. Of the 1427 who engaged with the online recruitment (clicked on the ads and provided parental consent and student assent), 1180 (83%) completed the survey. Of those, 953 responses (80%) were verified and were included in the analytic sample. Unfortunately, we do not have information about people who clicked on the link but did not obtain consent, because our IRB required that we delete that information. The final sample was 1690 respondents (737 school-based and 953 online).

### Statistical Analysis

We used *t*-tests and chi-squares to combine the school-based sample with the social media sample. Because the intraclass correlation of students nested within schools was very low (less than 0.05 for all variables) and because the social media participants were not clustered, we did not account for clustering in the analyses.

## Results

Table 1 shows the comparison of the school sample (*N* = 737) and the social media sample (*N* = 953).

**Table 1:** Comparison of adolescents recruited in schools vs. via social media

	School-based sample	Social media sample	Significance test
Age (mean [SD])	15.4 (1.1)	15.5 (1.2)	*t* = − 1.8 (*p* = 0.07)
Gender (N [%])			*X*^2^ = 9.1 (*p* = 0.01)
Male	359 (48.8%)	536 (56.9%)	
Female	376 (51.2%)	417 (43.8%)	
Grade in school			*X*^2^ = 357.1 (*p* < .0001)
9	285 (39%)	145 (15%)	
10	211 (29%)	204 (21%)	
11	235 (32%)	260 (27%)	
12	4 (1%)	190 (20%)	
Other/ungraded/not in school	0 (0%)	154 (16%)	
Race/ethnicity (N [%])			*X*^2^ = 355.4 (*p* < 0.001)
Hispanic/Latinx	3448 (60.8%)	170 (17.8%)	
White	136 (18.5%)	471 (49.4%)	
African American	51 (6.9%)	59 (6.2%)	
Asian/PI	67 (9.1%)	189 (19.8%)	
Other	35 (4.7%)	64 (6.7%)	
Language spoken at home			*X*^2^ = 135.9 (*p* < 0.001)
Only or Mostly English	362 (49.3%)	721 (75.7%)	
English and another language equally	209 (28.5%)	162 (17.0%)	
Only or Mostly another language	163 (22.2%)	70 (7.4%)	
Past-month substance use (N [%])			
Cannabis	124 (21.3%)	133 (17.9%)	*X*^2^ = 2.5 (*p* = 0.117)
Tobacco	117 (18.5%)	157 (17.4%)	*X*^2^ = 0.3 (*p* = .580)
Alcohol	241 (32.7%)	281 (29.5%)	*X*^2^ = 2.1 (*p* = 0.156)
Mental health (mean [SD])			
Depressive symptoms	19.8 (6.0)	21.2 (6.0)	*t* = 3.11 (*p* = .002)
Anxiety symptoms	12.8 (5.6)	14.1 (6.1)	*t* = 2.82 (*p* = .005)
Perceived stress	27.9 (6.9)	29.5 (6.8)	*t* = 3.17 (*p* = .001)

### Demographics

**Fig. 1:**
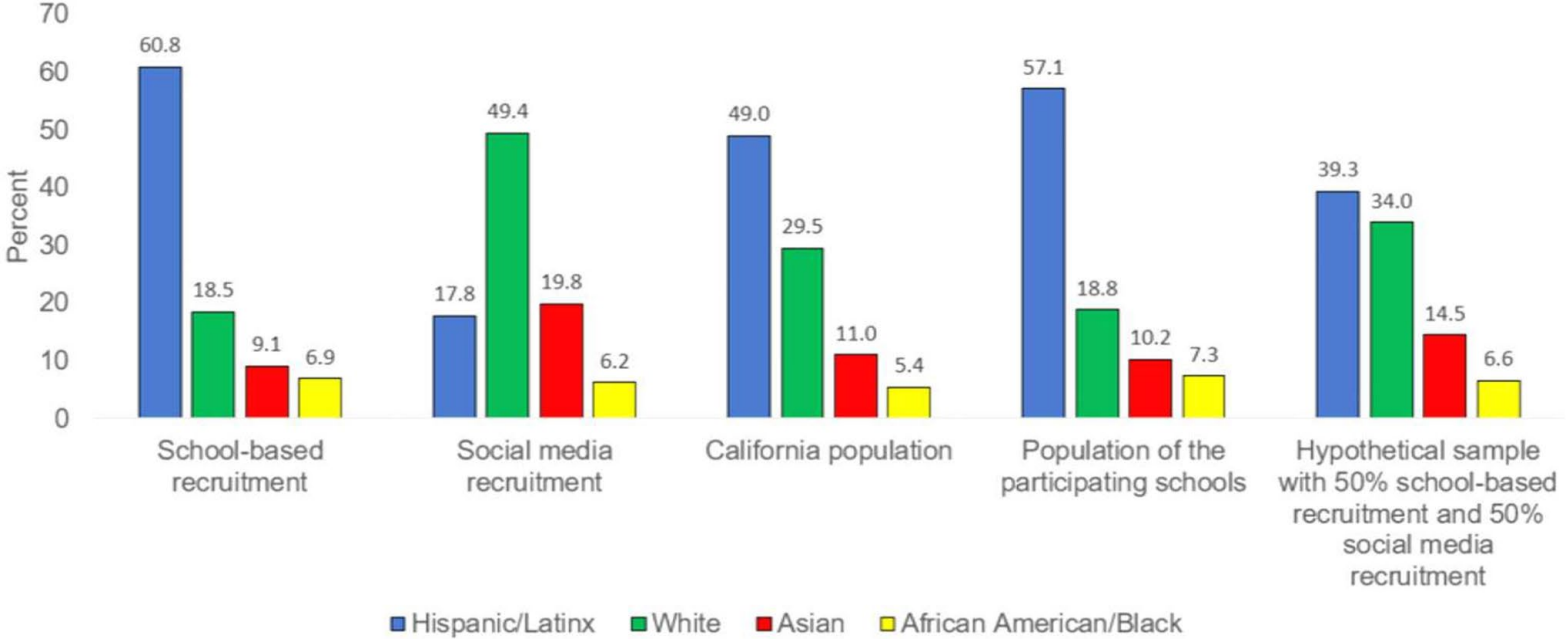
Racial/ethnic distribution of students recruited via schools vs. social media, compared with the California population and population of the participating schools

The two samples did not differ significantly on age. The social media sample was overrepresented by boys (57%), whereas the school-based sample had a more balanced gender distribution. Compared to the school-based sample, the social media sample was overrepresented by whites and Asians and underrepresented by Hispanic/Latinx. The proportion of African Americans was similar across the two samples. The social media sample had a higher proportion of students who spoke only or mostly English at home. Figure 1 shows the racial/ethnic composition of (1) the samples produced by each recruitment method; (2) the population of California youth ages 0–17 in 2020, (3) the underlying population of the participating schools, and (4) a hypothetical sample composed of 50% school-recruited adolescents and 50% social media-recruited adolescents. This hypothetical combined sample yielded a racial/ethnic distribution that was more similar to the California population than either recruitment method alone. The school-based sample was very similar in racial/ethnic distribution to the underlying population of the participating schools.

### Mental Health and Substance Use

Compared to the school-based sample, the adolescents in the social media sample reported higher levels of depressive symptoms, anxiety symptoms, and perceived stress. The two samples did not differ significantly on past-month use of cannabis, tobacco, or alcohol.

## Discussion

The abrupt closing of schools during the COVID-19 pandemic forced our research team to pivot from school-based data collection to social media data collection in the middle of an ongoing longitudinal study. Although changing data collection methods mid-study is not ideal, we were still able to recruit a large sample of adolescents that was approximately representative of the California population. In fact, a hypothetical sample composed of 50% school-recruited adolescents and 50% social media–recruited adolescents was more representative of the California population of adolescents than either recruitment method alone.

One drawback of the social media sample is that it recruited a smaller proportion of Hispanic/Latinx and students who spoke other languages at home, compared with school-based recruitment. Therefore, recruiting in public schools appears to be better for recruiting linguistically diverse samples.

It is interesting to note that although the two samples were different demographically, the adolescents’ self-reports of past-month substance use were similar. This suggests that social media recruitment could be a useful tool to estimate substance use prevalence, especially if samples could be weighted to represent the underlying population.

The social media sample reported more mental health symptoms (depression, anxiety, and stress) compared with the school sample. This is consistent with the finding that adolescents with mental health problems have more school absences and higher dropout rates (Allen et al., 2018; Esch et al., 2014). Adolescents with mental health problems also might have been diverted to alternative schools, which were not included in our school-based sample. Therefore, school-based samples are likely to miss the adolescents with the most severe mental health problems. However, it is also possible that the mental health problems reported by the social media-recruited students were a consequence of the COVID pandemic itself and the abrupt life changes that it caused just weeks before the adolescents were recruited. In addition, because adolescents with mental health problems are more likely to report heavy use of social media (Schønning et al., 2020), it is likely that the social media sample was overrepresented by adolescents with mental health problems.

### Limitations

The school-based sample was recruited right before the COVID-19 pandemic, and the social media sample was recruited during the pandemic. It is unclear whether the start of the pandemic altered adolescents’ mental health or substance use patterns during this tumultuous time. It is possible that the higher prevalence of mental health problems among the social media sample was a consequence of the pandemic itself, because the pandemic adversely affected mental health among adolescents (Samji et al., 2022). The low proportions of adolescents in the social media sample who were Hispanic/Latinx or spoke another language at home might have been due to the fact that the social media ads appeared in English only. The school-based consent forms were printed in English and Spanish. Future studies should place social media ads in multiple languages to reflect the multiple languages spoken by the underlying population.

Although social media recruitment can be easier than recruiting and visiting schools, it also had drawbacks. A social media sample will likely be overrepresented by heavy social media users, who tend to have a higher prevalence of mental health problems than the general population of adolescents (Schønning et al., 2020). Staff had to validate every participant to make sure they were not bots or scammers, and it is possible that some ineligible participants managed to evade our validation efforts by misreporting their ages or creating multiple email accounts to earn multiple gift cards. Researchers who recruit via social media should be alert to these limitations.

## Conclusion

Although school-based recruitment and social media recruitment produced samples with slightly different demographic characteristics, both samples yielded similar estimates of substance use prevalence. Social media recruitment could be an efficient strategy to recruit adolescents when school-based recruitment is not feasible. Social media recruitment could be an effective way to engage adolescents in research without disrupting schools, especially if the social media ads are targeted to more diverse audiences. A combination of school-based and social media recruitment methods could be used to overcome the selection biases inherent in each method. However, more research is needed to refine the procedures for obtaining representative samples via social media.

## Data Availability

Data are available from the authors.

## References

[CR1] Allen, C. W., Diamond-Myrsten, S., & Rollins, L. K. (2018). School absenteeism in children and adolescents. *American Family Physician,**98*(12), 738–744. 30525360

[CR2] Anderman, C., Cheadle, A., Curry, S., Diehr, P., Shultz, L., & Wagner, E. (1995). Selection bias related to parental consent in school-based survey research. *Evaluation Review,**19*(6), 663–674.

[CR3] Esbensen, F. A., Melde, C., Taylor, T. J., & Peterson, D. (2008). Active parental consent in school-based research: How much is enough and how do we get it? *Evaluation Review,**32*(4), 335–362. 18441216 10.1177/0193841X08315175

[CR4] Esch, P., Bocquet, V., Pull, C., Couffignal, S., Lehnert, T., Graas, M., & Ansseau, M. (2014). The downward spiral of mental disorders and educational attainment: A systematic review on early school leaving. *BMC Psychiatry,**14*, 1–13. 10.1186/s12888-014-0237-4 PMC4244046 25159271

[CR5] Gu, L. L., Skierkowski, D., Florin, P., Friend, K., & Ye, Y. (2016). Facebook, Twitter, & Qr codes: An exploratory trial examining the feasibility of social media mechanisms for sample recruitment. *Computers in Human Behavior,**1*(60), 86–96.

[CR6] Liu, C., Cox, R. B., Jr., Washburn, I. J., Croff, J. M., & Crethar, H. C. (2017). The effects of requiring parental consent for research on adolescents’ risk behaviors: A meta-analysis. *Journal of Adolescent Health,**61*(1), 45–52. 10.1016/j.jadohealth.2017.01.015 28363714

[CR7] Moreno, M. A., Waite, A., Pumper, M., Colburn, T., Holm, M., & Mendoza, J. (2017). Recruiting adolescent research participants: In-person compared to social media approaches. *Cyberpsychology, Behavior, and Social Networking,**20*(1), 64–67. 27976951 10.1089/cyber.2016.0319

[CR8] Plummer, B. D., Galla, B. M., Finn, A. S., Patrick, S. D., Meketon, D., Leonard, J., Goetz, C., Fernandez-Vina, E., Bartolino, S., White, R. E., & Duckworth, A. L. (2014). A behind-the-scenes guide to school-based research. *Mind, Brain, and Education,**8*(1), 15–20. 10.1111/mbe.12040 PMC4714562 26779282

[CR9] Reuter, K., Ukpolo, F., Ward, E., Wilson, M. L., & Angyan, P. (2016). Trial Promoter: A web-based tool for boosting the promotion of clinical research through social media. *Journal of Medical Internet Research,**18*(6), e144. 10.2196/jmir.4726. PMID: 27357424; PMCID: PMC4945821. 27357424 10.2196/jmir.4726 PMC4945821

[CR10] Rogers, C. J., Steinberg, J. K., Vos, R. O., Soto, D. W., & Unger, J. B. (2022). Associations between local jurisdiction ordinances and current use of cannabis products in California adolescents. *Substance Use and Misuse,**57*(3), 373–379. 10.1080/10826084.2021.2012693. Epub 2021 Dec 14 PMID: 34903134. 34903134 10.1080/10826084.2021.2012693

[CR11] Samji, H., Wu, J., Ladak, A., Vossen, C., Stewart, E., Dove, N., Long, D., & Snell, G. (2022). Mental health impacts of the COVID-19 pandemic on children and youth–A systematic review. *Child and Adolescent Mental Health,**27*(2), 173–189. 34455683 10.1111/camh.12501 PMC8653204

[CR12] Schønning, V., Hjetland, G. J., Aarø, L. E., & Skogen, J. C. (2020). Social media use and mental health and well-being among adolescents–A scoping review. *Frontiers in Psychology,**14*(11), 1949. 10.3389/fpsyg.2020.01949 PMC7457037 32922333

[CR13] Stern, M. J., Fordyce, E., Hansen, C., Heim Viox, M., Michaels, S., Schlissel, A., Avripas, S., Harper, C., Johns, M., & Dunville, R. (2020). Social media recruitment for a web survey of sexual and gender minority youth: An evaluation of methods used and resulting sample diversity. *LGBT Health,**7*(8), 448–456. 33147121 10.1089/lgbt.2019.0311

[CR14] Unger, J. B., Gallaher, P., Palmer, P. H., Baezconde-Garbanati, L., Trinidad, D. R., Cen, S., & Johnson, C. A. (2004). No news is bad news: Characteristics of adolescents who provide neither parental consent nor refusal for participation in school-based survey research. *Evaluation Review,**28*(1), 52–63. 14750291 10.1177/0193841X03254421

[CR15] Vannucci, A., Simpson, E. G., Gagnon, S., & Ohannessian, C. M. (2020). Social media use and risky behaviors in adolescents: A meta-analysis. *Journal of Adolescence,**79*, 258–274. 10.1016/j.adolescence.2020.01.014. Epub 2020 Feb 1 PMID: 32018149. 32018149 10.1016/j.adolescence.2020.01.014

[CR16] Vidal, C., Lhaksampa, T., Miller, L., & Platt, R. (2020). Social media use and depression in adolescents: A scoping review. *Int Rev Psychiatry*, *32*(3), 235–253. 10.1080/09540261.2020.1720623. Epub 2020 Feb 17. PMID: 32065542; PMCID: PMC7392374. 10.1080/09540261.2020.1720623 PMC7392374 32065542

[CR17] Weitzman, B. C., Guttmacher, S., Weinberg, S., & Kapadia, F. (2003). Low response rate schools in surveys of adolescent risk taking behaviours: Possible biases, possible solutions. *Journal of Epidemiology & Community Health,**57*(1), 63–67. 12490651 10.1136/jech.57.1.63 PMC1732264

